# Contribution to diagnosis and treatment of bone marrow aspirate results in critically ill patients undergoing bone marrow aspiration: a retrospective study of 193 consecutive patients

**DOI:** 10.1186/s40560-017-0263-7

**Published:** 2017-12-04

**Authors:** Laure Calvet, Bruno Pereira, Anne-Françoise Sapin, Gabrielle Mareynat, Alexandre Lautrette, Bertrand Souweine

**Affiliations:** 1 Service de Réanimation Médicale, Hôpital Gabriel Montpied, CHU de Clermont-Ferrand, BP 69, 63003 Clermont-Ferrand, Cedex 1, France; 2 Département de biostatistique, CHU de Clermont-Ferrand, Clermont-Ferrand, France; 3 Laboratoire d’hématologie, CHU de Clermont-Ferrand, Clermont-Ferrand, France; 4 Université Clermont Auvergne, CNRS, LMGE, F-63000 Clermont-Ferrand, France

**Keywords:** Bone marrow, Hemophagocytic lymphohistiocytosis, Thrombocytopenia, Critically ill patient

## Abstract

**Background:**

The purpose of the work was to assess the contribution to diagnosis and/or treatment (CDT) of bone marrow aspiration (BMA) in the critically ill patient.

**Methods:**

The retrospective study included 193 patients. On the basis of BMA findings, contribution to diagnosis was defined by one of four previously unestablished diagnoses (maturation arrest of granulocyte precursors, hemophagocytic lymphohistiocytosis, hematological malignancy, marrow infiltration with cancer cells) and to treatment as the initiation or withdrawal of a specific treatment including the decision to forgo life-sustaining treatment (DFLST).

**Results:**

A CDT of BMA was observed in 40/193 patients (20.7%). BMA contributed to diagnosis in 37 cases (granulocyte precursor maturation arrest, *N* = 10; hemophagocytic lymphohistiocytosis, *N* = 12; hematological malignancy, *N* = 15) and to treatment in 14, including three DFLSTs. In multivariate analysis, the factors associated with a CDT were hematological malignancy, cancer or non-malignant hematological abnormality known on admission, indication for BMA excluding isolated thrombocytopenia, higher pre-BMA HScore (calculated prior to BMA), and higher SOFA score with or without platelet-count SOFA subscore. In the 160 patients without hematological malignancy or cancer known on admission, non-malignant hematological abnormality known on admission, indication for BMA excluding isolated thrombocytopenia, higher pre-BMA HScore, and higher SOFA score calculated with or without platelet-count SOFA subscore were independently associated with a CDT of BMA.

**Conclusion:**

BMA can have a significant CDT in ICU patients with or without a known hematological malignancy or cancer on admission. An HScore calculated before BMA can be a valuable tool for predicting a CDT of BMA.

**Electronic supplementary material:**

The online version of this article (10.1186/s40560-017-0263-7) contains supplementary material, which is available to authorized users.

## Background

Bone marrow aspiration (BMA) is mainly performed for cytomorphological examination of bone marrow cells, but also to proceed to other analyses such as immunophenotypic, flow cytometry, cytogenetic, molecular genetic, and microbiological tests. BMA is an important medical procedure for the diagnosis, staging, and follow-up of patients with hematological diseases and for investigating various non-hematological conditions including storage diseases, inborn errors of metabolism, metastatic cancer, and infection that has spread to the bone marrow.

BMA is easy to perform, and its examination often provides a reliable diagnosis within a matter of hours. However, it is invasive and painful requiring the administration of local anesthesia in patients not receiving general anesthetic sedation. Adverse events following BMA are rare but may result in severe complications [1, 2]. The indications for BMA are established in routine hematology practice [3].

Few reports have attempted to measure the contribution to diagnosis and/or treatment (CDT) of BMA in intensive care unit (ICU) patients, and most available data come from cohort studies of subgroups of patients with thrombocytopenia [4–6] or hemophagocytic lymphohistiocytosis (HLH) [7–9]. In ICU patients, BMA can be indicated for numerous conditions: as part of the follow-up of a malignancy previously known upon admission, for diagnostic purposes, and to guide treatment in patients with unexplained clinical features or laboratory or radiologic abnormalities such as lymphadenopathy, hepato-splenomegaly, osteolytic bone lesions, hypercalcemia, monoclonal proteins, cytopenia, cytosis, and the presence of immature or morphologically atypical cells in the peripheral blood. Dealing with these factors in the ICU is particularly difficult since these medical circumstances often result from multiple mechanisms caused by the severity of the acute illness, the underlying conditions, and their respective treatments. Because of the lack of information on BMA findings in the critically ill, the indications for the procedure are not standardized in the ICU and are highly dependent on local hospital practice and available technical expertise.

The aim of this retrospective study was therefore to assess the results of BMA performed in ICU patients and to determine the CDT of BMA in this subpopulation.

## Methods

### Setting and population

This retrospective study was carried out in the 10-bed medical ICU of the University Hospital of Clermont-Ferrand (France). All consecutive adult patients (age > 18 years) who underwent a BMA between 1 January 2010 and 31 October 2014 were screened. They were identified by electronic search in the database of the hematology department. Patients with no adequate bone marrow specimen because BMA had resulted in a dry tap or very dilute sample were excluded from the study. For patients with multiple ICU admissions over the study period, only the first ICU stay with a BMA yielding an adequate specimen was included in the analysis. For patients who had multiple adequate BMA specimens during the same ICU stay, only the first adequate specimen was taken into account in the analysis. At our institution, senior hematologists and intensivists are available 24 h a day 7 days a week and work together to indicate BMA. BMA is ordered to make a specific diagnosis, guide specific treatment, withhold potentially ineffective and/or harmful treatment, or provide important prognostic information. The data collected from the medical records are given in Additional file 1 [10–12] and the procedure of BMA in Additional file 2. The HScore was calculated for each patient before (pre-BMA HScore) and after (HScore) BMA results. The HScore is the first validated score to estimate individual risk of HLH. It was calculated from variables defined by a web-based Delphi study. The weight of each of the criteria was established by logistic regression modeling. The HScore is based on a set of nine weighted clinical, biological, and cytologic criteria. The minimum and maximum values of the pre-BMA HScore (before BMA results) are 0 and 302 points, respectively. The minimum and maximum values of the HScore are 0 and 337 points, respectively. The best diagnostic threshold of the HScore for HLH was 169 with a probability of HLH of 52%, corresponding to a sensitivity of 93%, a specificity of 86%, and an accurate classification of 90% of the patients [13].

Hematologists with knowledge of all clinical, radiologic, and laboratory tests analyze BMA samples. This study was approved by our institutional review board (Institutional review Board of Clermont-Ferrand South-East 6 – IRB00008526 number 2016/CE51) in accordance with French regulations. No consent was needed from patients.

### Definitions

The indications for BMA were divided into agranulocytosis, isolated thrombocytopenia, and suspicion of hemophagocytic lymphohistiocytosis (HLH), of hematological malignancy, and of cancer that had spread to the bone marrow. Agranulocytosis was considered as the indication for BMA when it was not associated with another indication. Isolated thrombocytopenia was considered as the indication for BMA when thrombocytopenia was the only indication for BMA. An adjudication committee (LC and BS) determined whether BMA made a CDT. Contribution to diagnosis was defined as a BMA result pointing to one of the following previously unestablished diagnoses: maturation arrest of granulocyte precursors, HLH as defined by HScore calculation threshold ≥ 169 [13], hematological malignancy, and infiltration with cancer cells. Contribution to therapy was defined as the initiation or discontinuation of a specific therapy strategy based on BMA findings, and the decision to forgo life-sustaining treatment (DFLST). Pre-BMA HScore was defined as HScore calculated with no points assigned for the variable “hemophagocytosis features on bone marrow aspirate.” Post-BMA complications were bleeding or infection at the site of aspiration and other severe adverse events related to sternal aspiration such as manubrial separation and cardiac tamponade.

### Statistical analysis

Statistical analysis was performed with Stata software (version 13, StataCorp, TX). All tests were performed for a two-tailed type I error at 0.05. Quantitative data were expressed as mean ± standard deviation or median [interquartile range]. CDT was expressed in percentages (number of patients with a BMA yielding a CDT divided by number of patients in the study). Between-group comparisons were performed by chi-squared or Fisher’s exact tests and Student’s *t* test or Mann-Whitney test if *t* test conditions were not met. Relationships between quantitative parameters were studied by Pearson or Spearman correlation coefficients. Multivariable logistic regression was performed to predict CDT, with covariables determined according to univariate results and their clinical relevance. The results were expressed as odds ratios and 95% confidence intervals. The diagnostic accuracy of the pre-BMA HScore in predicting the CDT of BMA was evaluated with the area under the receiver-operating characteristic (AUC-ROC) curve. The optimal threshold was determined according to standard indices (Liu, Youden, and efficiency). A sensitivity analysis was performed on the subpopulation.

## Results

### Characteristics of patients and CDT of BMA

**Fig. 1:**
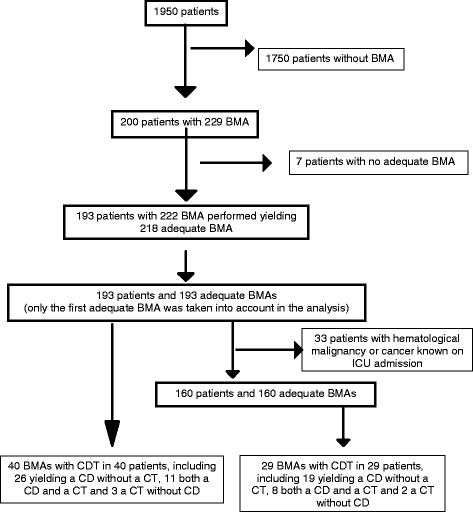
Flow chart of the study. BMA, bone marrow aspiration; CD, contribution to diagnosis, CDT, contribution to diagnosis and/or treatment; CT, contribution to treatment; ICU, intensive care unit

**Fig. 2:**
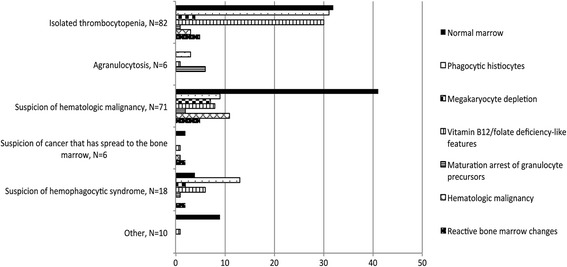
Indications for bone marrow aspiration and pathological: Megakaryocyte depletion, one or fewer megakaryocytes per 5 to 10 low-power fields on bone marrow examination [15] Other, suspicion of tuberculosis (*N*=5), undetermined (*N*=5); Reactive bone marrow changes, non-specific bone marrow modifications associated with acute inflammation including hypercellularity, an increased myeloid-to-erythroid ratio with a large number of myeloid precursors and mature segmented neutrophils, with or without megakaryocyte hyperplasia, monocytosis and a slight increase in normal plasmocytes [14]; Vitamin B12/folate deficiency -like features, cytological signs classically associated with vitamin B12/folate deficiency including hypersegmented neutrophil granulocytes, giant metamyelocytes, erythroid asynchronism maturation

During the study period, 193 patients fulfilled the inclusion criteria (Fig. 1). The annual rate of ICU patients admitted with an adequate BMA did not differ over time (*p* = 0.39; Additional file 3). The characteristics of the study population are given in Table 1. The results of blood count and coagulation tests on the day of BMA are given in Additional file 4. The indications for, and pathological findings of, BMA are shown in Fig. 2 [14, 15]. BMA had a CDT in 40 patients (20.7%). It contributed to diagnosis in 37 patients and to treatment in 14 (Table 2). In the 10 patients with maturation arrest of granulocyte precursors, exposure to potentially hematotoxic agents within the preceding 7 days was identified in all cases, but corresponded to an immunosuppressive treatment in only one case (Additional file 5). Of the 12 patients with HLH, 7 were severely immunocompromised. Infection was the precipitating factors in 10 patients including herpesviridae infection in 5.

**Table 1:** Characteristics of the study population

Patients	*n* = 193
Baseline characteristics	
Age (years)^a^	66 ± 14
Gender ratio (male/female)	1.5
Body mass index^a^	26 ± 7
SOFA on ICU admission^b^	7 [5–11]
SAPS II on ICU admission^b^	60 [40–72]
McCabe score 2^c,d^	26 (13)
Hematological malignancy or cancer previously diagnosed^c^	33 (17)
Nonmalignant hematological abnormality previously diagnosed^c,e^	14 (7)
Reason for ICU admission^c^	
Acute respiratory failure	67 (35)
Severe sepsis/septic shock	50 (26)
Acute renal failure and metabolic disorder	28 (14)
Coma	19 (10)
Other^f^	29 (15)
Vitamin B12 deficiency^c,g,h^	2 (2)
Vitamin B9 deficiency^c,g,i^	25 (36)
Days from admission to biopsy (days)^b^	5 [3–8]
Characteristics on the day of BMA	
BMA at the sternal site^c^	155 (80)
BMA at the iliac crest site	38 (20)
Sepsis^cj^	124 (64)
Adenopathy^c,j^	14 (7)
Splenomegaly^c^	7 (4)
Monoclonal gammapathy^c^	18 (9)
Anticoagulant agent^c,k^	129 (67)
Prophylactic anticoagulation^c^	87 (45)
Therapeutic anticoagulation^c^	42 (22)
Antiplatelet agent^c^	31 (16)
Proton-pump inhibitor agent^c^	40 (21)
Anti-infectious agent with potential hematoxcicity^c^	146 (76)
Other agent with potential hematoxcicity^c^	76 (39)
Pre-BMA HScore	62 [24–96]
SOFA^c^	8 [5–11]
Invasive ventilation^c^	52 (27)
Vasopressors^c^	90 (47)
Renal replacement therapy	31 (16)
ICU length of stay^b^	14 [7–29]
ICU mortality^c^	59 (31)
Hospital mortality^c^	78 (40)

*ICU* intensive care unit; *PreBMA HScore*, *SAPS II* simplified acute physiology score, *SOFA* Sequential Organ Failure Assessment score

^a^Mean ± SD

^b^Median [interquartile range]

^c^Number (percentage)

^d^Fatal outcome within 12 months

^e^Thrombocytopenia (*n* = 5), monoclonal gammopathy of undetermined significance (*n* = 3), polycythemia (*n* = 2), auto-immune cytopenia (*n* = 4)

^f^Cardiac arrest (*N* = 9), other shock (*N* = 8), thrombotic microangiopathy (*N* = 7), post-surgery surveillance (*N* = 5)

^g^Blood samples drawn between admission and BMA

^h^Eighty-two missing data

^i^One hundred twenty-four missing data

^j^In 48 patients, sepsis was unresolved since ICU admission

^k^Unfractionated heparin (*N* = 44), low-molecular-weight heparin (*N* = 78), danaparoid (*N* = 7)

**Table 2:** Contribution of bone marrow aspiration to diagnosis and treatment

Patient	Indication	Diagnostic contribution, *N* = 37	Therapeutic contribution, *N* = 14
1	b	Hemophagocytic syndrome	No
2 (a)	b	Maturation arrest of granulocyte precursors	No
3 (a)	c	Acute transformation of CMML	DFLST
4	d	Maturation arrest of granulocyte precursors	No
5 (a)	c	Maturation arrest of granulocyte precursors	No
6	c	No (normal marrow)	Addition of erythropoietin
7	c	Marginal zone lymphoma	No
8 (a)	c	No (normal marrow)	Addition of dasatinib
9 (a)	e	Maturation arrest of granulocyte precursors	Discontinuation of TMP/SMX
10	e	Hemophagocytic syndrome	No
11	c	Lymphocytic lymphoma	No
12	e	No (presence of phagocytic histiocytes)	Addition of etoposide
13	e	Myelodysplastic syndrome	No
14	b	Hemophagocytic syndrome	No
15	c	Diffuse large B cell lymphoma	Addition of COP (g)
16	c	Marginal zone lymphoma	No
17	d	Maturation arrest of granulocyte precursors	No
18 (a)	c	Acute monocytic leukemia	DFLST
19	c	Acute myeloid leukemia	DFLST
20	e	Hemophagocytic syndrome	No
21	e	Hemophagocytic syndrome	No
22	c	Myelodysplastic syndrome	No
23 (a)	c	Relapsed multiple myeloma	No
24	e	Hemophagocytic syndrome	No
25	d	Maturation arrest of granulocyte precursors	Discontinuation of tacrolimus
26	e	Hemophagocytic syndrome	No
27 (a)	c	Hemophagocytic syndrome	No
28	c	Multiple myeloma	No
29	e	Myelodysplastic syndrome	No
30	e	Myelodysplastic syndrome	No
31	b	Hemophagocytic syndrome	No
32	f	Diffuse large B cell lymphoma	Addition of COP (g)
33 (a)	c	Myelodysplastic syndrome	No
34	e	Hemophagocytic syndrome	Addition of etoposide
35 (a)	c	Hemophagocytic syndrome	No
36	e	Hemophagocytic syndrome	No
37	d	Maturation arrest of granulocyte precursors	Addition of lenogastrim
38	d	Maturation arrest of granulocyte precursors	Discontinuation of amoxicillin
39	d	Maturation arrest of granulocyte precursors	Addition of lenogastrim
40 (a)	c	Maturation arrest of granulocyte precursors	No

In patients 6, 8, and 12, BMA did not contribute to diagnosis but contributed to treatment

a, hematological malignancy or cancer already known upon ICU admission; b, suspicion of hemophagocytic lymphohistiocytosis; c, suspicion of hematological malignancy; d, agranulocytosis; e, thrombocytopenia; f, suspected cancer that has spread to the bone marrow; g, ultimately followed by DFLST; *CMML*, chronic myelomonocytic leukemia; *COP*, cyclophosphamide, vincristine, prednisone; *DFLST*, decision to forego life-sustaining treatment; *TMP/SMX*, trimethoprim/sulfamethoxazole

In the 14 patients with a BMA contributing to treatment, BMA findings resulted in initiation of treatment in 8, discontinuation in 3, and a DFLST in the other 3, established on the basis of the reported diagnosis (acute myeloid leukemia) against a background of worsening critical state (Table 2). No post-BMA complications were observed.

### Factors associated with BMA yielding a CDT

In the overall population, the factors associated with a CDT of BMA in univariate analysis are given in Additional file 6. In multivariate analysis, the factors significantly associated with a CDT of BMA were a known diagnosis on ICU admission of either cancer or hematological malignancy or non-malignant hematological abnormality; indication for BMA excluding isolated thrombocytopenia; higher pre-BMA HScore; and higher SOFA score calculated with or without platelet subscore on the day of BMA (Table 3). The AUC-ROC curve of pre-BMA HScore that predicted the CDT of BMA was 0.76 (95% confidence interval = 0.66–0.85). Youden’s Index was maximal for a threshold of 76 with a sensitivity of 68% and a specificity of 73%. In a sensitive analysis performed in the subpopulation of 181 patients without HLH, a higher pre-BMA HScore was independently associated with a CDT of BMA (Additional file 7). CDT was more often observed in patients with hematological malignancy or cancer known on admission than in patients with no hematological malignancy or cancer known on admission (29/160 (18%) vs 11/33 (33%), *p* = 0.05).

**Table 3:** Multivariable analysis of factors associated with a CDT of BMA in the overall population (*N* = 193)

Variable	Odds ratio	95% confidence interval	*P* value
First model			
Hematological malignancy, cancer, or non-malignant hematological abnormality known on admission	3.70	[1.44–9.33]	0.007
Indication of BMA excluding isolated TP^a,b^	3.69	[1.46–9.30]	0.006
SOFA score^c^	1.15	[1.04–1.28]	0.006
Pre-BMA HScore^c,d^	1.03	[1.02–1.04]	< 0.001
Second model			
Hematological malignancy, cancer, or non-malignant hematological abnormality known on admission	3.21	[1.78–8.76]	0.023
Indication of BMA excluding isolated TP^a,b^	4.53	[1.66–12.38]	0.003
SOFA—platelet-count SOFA subscore^c^	1.15	[1.03–1.29]	0.013
Pre-BMA HScore^c,d^	1.03	[1.02–1.04]	< 0.001
Platelet count SOFA subscore 0 versus other groups^c^	2.38	[0.76-7.50]	0.138

*BMA* bone marrow aspiration, *CDT* contribution to diagnosis and/or treatment, *Hscore* reactive hemophagocytic syndrome diagnostic score, *SOFA* sequential organ failure assessment, *TP* thrombocytopenia

^a^Thrombocytopenia may be present or absent in these patients

^b^Isolated thrombocytopenia, i.e., thrombocytopenia was the only indication for BMA

^c^Per point

^d^Calculated with no points assigned for the cytological variable

In the 160 patients without hematological malignancy or cancer known on admission, the factors associated with a CDT of BMA in univariate analysis are given in Additional file 8. In multivariate analysis, the factors significantly associated with a CDT of BMA were as follows: a non-malignant hematological abnormality known on admission, indication of BMA excluding isolated thrombocytopenia, higher pre-BMA Hscore, and higher SOFA score calculated with or without platelet-count SOFA subscore on the day of BMA (Table 4). The AUC-ROC curve of pre-BMA HScore that predicted the CDT of BMA was 0.75 (95% confidence interval = 0.64–0.86). Youden’s Index was maximal for a threshold of 76 with a sensitivity of 69% and a specificity of 74%.

**Table 4:** Multivariable analysis of factors associated with a CDT of BMA in the subpopulation with no hematological malignancy or cancer known on admission (*N* = 160)

Variable	Odds ratio	95% confidence interval	*P* value
First model			
Non-malignant hematological abnormality known on admission	7.75	[1.77–34.02]	0.007
Indication of BMA excluding isolated TP^a,b^	3.32	[1.16–9.51]	0.025
SOFA score^c^	1.19	[1.06–1.34]	0.003
Pre-BMA HScore^c,d^	1.03	[1.01–1.04]	< 0.001
Second model			
Non-malignant hematological abnormality known on admission	6.76	[1.51–30.38]	0.013
Indication of BMA excluding isolated TP^a,b^	3.82	[1.19–12.24]	0.024
SOFA—platelet-count SOFA subscore^c^	1.19	[1.04–1.35]	0.009
Pre-BMA HScore^c,d^	1.03	[1.01–1.04]	< 0.001
Platelet count SOFA subscore 0 versus other groups	2.14	[0.60–7.62]	0.239

*BMA* bone marrow aspiration, *CDT* contribution to diagnosis and/or treatment, *Hscore* reactive hemophagocytic syndrome diagnostic score, *SOFA* sequential organ failure assessment, *TP* thrombocytopenia

^a^Thrombocytopenia may be present or absent in these patients

^b^Isolated thrombocytopenia, i.e., thrombocytopenia was the only indication for BMA

^c^Per point

^d^Calculated with no points assigned for the cytological variable

## Discussion

This study shows that in 21% of our ICU patients undergoing BMA, the results of the examination had a significant impact on diagnosis and management. We found a higher CDT in patients with higher values of SOFA scores calculated with or without platelet count subscore, in patients with higher values of pre-BMA HScore, and in patients with indication for BMA not restricted to isolated thrombocytopenia. These results were observed in our study population in patients both with and without hematological malignancy or cancer known on ICU admission.

Data on BMA in ICU patients are scant, and studies on this topic have focused on specific subpopulations, mostly of patients with thrombocytopenia [4–6] or with HLH [7–9]. To the best of our knowledge, our study is the first to report the CDT of bone marrow examination in an overall adult ICU population undergoing BMA for any reason.

The etiologies of thrombocytopenia in the ICU setting are classically multifactorial and mainly due to increased peripheral platelet destruction [16–18]. In a multicenter study involving 301 consecutive thrombocytopenic ICU patients, BMA findings yielded a previous unestablished diagnosis in 22% of patients and prompted a change in therapeutic management in 11% [6]. In our study, thrombocytopenia was present at the time of BMA in 128 patients and yielded a contribution to diagnosis in 32 (25%) and to treatment in 10 (8%). We found a higher CDT in patients undergoing BMA for indications excluding isolated thrombocytopenia. This suggests that although cases of thrombocytopenia are currently due to multiple etiologies in the ICU setting, BMA should not be systematically performed on the basis of a single low platelet count. The decision to carry out a BMA in thrombocytopenic critically ill patients should include the presence of other features such as a detailed clinical history, physical examination, review of all medications, and results of other diagnostic procedures including blood test coagulation, blood cell counts, and peripheral blood smear examination.

Reactive HLH is a life threatening disorder. Its course may be improved by early etoposide administration. Several studies have reported the results of BMA in adult ICU patients with reactive HLH but used different definitions, which makes it difficult to compare their findings [7–9]. In our study, HLH was defined by the recently developed HScore [13], which includes variables defined by a web-based Delphi study [19], with the weight of each of the criteria established by logistic regression modeling.

The presence of hemophagocytosis on BMA specimens is not pathognomonic of HLH [8, 13] and is found, for instance, in severely ill patients with sepsis or after blood transfusion but with no proven HLH [7–9, 13, 20, 21]. In our study, hemophagocytosis on BMA examination was observed in 56 (29%) patients but corresponded to HLH in only 12. In addition, hemophagocytic activity may be absent during HLH particularly at the initial phases [22]. In a retrospective study performed in a medical ICU with HLH diagnosed according to criteria established by the International Histiocyte Society revised in 2004 [23], histological evidence of hemophagocytosis was not observed in 12/56 patients (22%) with HLH [8]. In a recent study performed in 98 adult ICU patients with BMA requested for suspicion of HLH and HLH diagnosed on an HScore threshold value higher than 169, hemophagocytosis on BMA examination was observed in 57/71 (83%) patients with HLH and in 21/27 (84%) without [9]. In our study, 12 patients had HLH defined by an HScore threshold value higher than 169, and hemophagocytosis on BMA examination was observed in all 12 cases. Infection was the predominant precipitating factor, as classically reported [22]. We found that a higher pre-BMA score was associated with a CDT of BMA even in our subpopulation without HLH. This likely reflects the inclusion in the HScore of clinical and laboratory manifestations such as known underlying immunosuppression, fever, organomegaly (splenomegaly, hepatomegaly), and cytopenias, which were also commonly associated with diagnoses other than HLH defining a CDT of BMA in our study (maturation arrest of granulocyte precursors, hematological malignancy, and marrow infiltration with cancer cells). Thus, our study suggests that a single score, the HScore calculated either before BMA (pre-BMA HScore) or after, could be a useful means of both predicting a CDT of BMA and establishing HLH diagnosis.

The incidence of BMA-related mechanical complications reported in the literature is lower than 0.4% [2, 6, 24, 25]. In our study, we observed no BMA procedure-related complications, which is in agreement with the results of a previous report [6]. We are aware that our study has several limitations. First, it was retrospective. Second, it involved only ICU patients undergoing BMA, and hence, the CDT of BMA in a general ICU population cannot be extrapolated from our results. Third, it was a single-center study performed in a medical ICU, and whether the results can be extrapolated to other ICUs remains questionable. Fourth, the indications for BMA were not pre-defined, although the year-on-year rates of patients who had a BMA did not differ over the period studied. Fifth, the definition of “contribution to diagnosis” did not account for factors such as the mechanism of thrombocytopenia or signs suggesting vitamin deficiency. The mechanism of thrombocytopenia was intentionally ruled out of the definition as all of the bone marrow examinations ordered to screen thrombocytopenia would have necessarily been diagnosis-contributive in some way given the “yes/no” output (absence or presence of megakaryocyte depletion). Likewise, the presence on bone marrow examination of signs pointing to vitamin B9 or B12 deficiency was not factored into the definition of contribution to diagnosis as signs like these are frequently found on ICU-patient aspirates yet only rarely associated with an actual deficit as evidenced in biological screening assays [6, 26]. Sixth, the definition of contribution to diagnosis did not include ongoing treatment when BMA yielded no abnormal findings. Normal BMA findings in a cytopenic patient can serve to rule out any potential myelotoxicity from certain drug therapies and thus allow them to continue. As our study was retrospective, we were unable to run this type of analysis. The latter two points may mean that we underestimated the CDT of the bone marrow examination here. In the intensive care setting, BMA is useful in cases of unexplained cytopenia, suspected HLH, suspected cancer that has spread to the bone marrow, or suspected hematological malignancy. The decision to perform a BMA in the ICU should be taken by a multidisciplinary team approach including intensivists and hematologists, and it requires the integration of various factors including clinical history, physical examination, peripheral blood film analysis, and other diagnostic procedure results, as recommended in other medical units [27]. In these conditions, BMA in the intensive care setting can yield the diagnosis of maturation arrest of granulocyte precursors, HLH or hematological malignancy, and bone marrow infiltration with cancer cells and also help in the decision to initiate or discontinue a specific therapy strategy. In some cases such as lymphomas, myeloproliferative neoplasms, or metastatic malignancies, BMA cannot always establish the diagnosis and should then be combined with a trephine biopsy.

## Conclusion

BMA can be a valuable tool in critical care management, in patients both with and without hematological malignancy or cancer known on admission. The HScore may help to better define the population liable to benefit from BMA. Multicenter cohort studies should be performed to confirm these results and to determine the usefulness of BMA in an overall ICU population.

## Additional files

Additional file 1: Data collection. (DOCX 14 kb)

Additional file 2: Bone marrow aspiration procedures. (DOCX 11 kb)

Additional file 3: Annual rates of ICU admissions and adequate bone marrow aspirations. (DOCX 48 kb)

Additional file 4: Hematological parameters on the day of bone marrow aspiration in the overall population (*N* = 193) and in patients with BMA results yielding a CDT (*N* = 40). (DOCX 12 kb)

Additional file 5: Hematotoxic agents administered within the 7 days prior to bone marrow aspiration in the 10 patients with maturation arrest of granulocyte precursors observed on marrow aspirates. (DOCX 13 kb)

Additional file 6: Results of the univariate analysis in the overall population (*n* = 193 patients). (DOCX 14 kb)

Additional file 7: Multivariable analysis of factors associated with a CDT of BMA in the 181 patients without HLH. (DOCX 12 kb)

Additional file 8: Results of the univariate analysis in the 160 patients without hematological malignancy or cancer known on admission. (DOCX 14 kb)

## Abbreviations

AUC-ROC: Area under the receiver-operating characteristic
BMA: Bone marrow aspiration
CDT: Contribution to diagnosis and/or treatment
DFLST: Decision to forgo life-sustaining treatment
HLH: Hemophagocytic lymphohistiocytosis
ICU: Intensive care unit
SOFA: Sequential organ failure assessment
