# Supplementary material for: Contribution to diagnosis and treatment of bone marrow aspirate results in critically ill patients undergoing bone marrow aspiration: a retrospective study of 193 consecutive patients
Source: J Intensive Care. 2017 Dec 4;5:67. doi: 10.1186/s40560-017-0263-7 (PMC5715543; doi:10.1186/s40560-017-0263-7)
Supplement: Supplementary file 1 — Data collection. (DOCX 14 kb) [file 40560_2017_263_MOESM1_ESM.docx]

Additional file 1, Data collection

Data extracted from the medical records were demographic characteristics, reason for ICU admission, underlying diseases, McCabe score (10), sequential organ failure assessment (SOFA) score (11), Simplified Acute Physiology Score (SAPS II)(12) on admission, drug exposure in the 7 days prior to BMA with a special emphasis on antibiotics, antivirals, antifungals, immunosuppressive and antineoplastic agents, furosemide, proton pump inhibitors, antiplatelets, heparin and anticonvulsants, serum levels of vitamin B12 and folic acid when measurements were performed before BMA, supportive care during ICU stay, ICU length of stay, and ICU and hospital mortality. The following data were collected on the day of BMA: time from admission, presence of sepsis, higher temperature measurement, SOFA score, laboratory tests, including full blood count and peripheral blood smear, coagulation parameters and serum level measurement of ferritin, triglyceride, aspartate aminotransferase (ASAT), and alanine aminotransferase (ALAT). We also recorded the BMA technique, marrow aspirate findings, HScore (13), post-BMA therapeutic changes including decisions to forgo life-sustaining treatment (DFLST), and post-BMA complications.
