# Supplementary material for: Contribution to diagnosis and treatment of bone marrow aspirate results in critically ill patients undergoing bone marrow aspiration: a retrospective study of 193 consecutive patients
Source: J Intensive Care. 2017 Dec 4;5:67. doi: 10.1186/s40560-017-0263-7 (PMC5715543; doi:10.1186/s40560-017-0263-7)
Supplement: Supplementary file 2 — Bone marrow aspiration procedures. (DOCX 11 kb) [file 40560_2017_263_MOESM2_ESM.docx]

Additional file 2, Bone marrow aspiration procedures

BMA was bedside performed by trained operators using standard technique. The preferred anatomic site for BMA was the sternal site. The iliac crest was used when sternal aspiration was contraindicated (patient with prior sternal fracture, sternotomy or mediastinal radiotherapy), and when bone marrow trephine was also indicated, and when BMA at sternal site had previously failed. All procedures used 16G/1.6 mm-diameter disposable needles (Thiebaud Biomedical Devices, Thonon-les-Bains, France) with a length of 20 mm to 30 mm for sternal aspiration and of 50 mm for iliac aspiration. Thrombocytopenia and coagulation abnormalities were considered a contraindication to BMA at the iliac crest site but not at the sternal site. The procedure was performed under sterile conditions, which comprise skin antisepsis with alcoholic chlorhexidine, wearing cap, mask, sterile gown and gloves, and placing a sterile drape with a fenestrated opening over the procurement site. In patients not receiving systemic analgesics, a local anesthesia with lidocaine was used to numb the skin and periosteum at the site of marrow procurement. Approximately an aspirate of one mL was drawn with a 10- or 20-ml anticoagulant-free plastic syringe attached to the aspiration needle. The marrow sample was immediately placed into a tube containing EDTA anticoagulant, gently shaken and promptly sent to the hematology laboratory for smear preparation. Smears were prepared from the aspirated material upon arrival at the laboratory and stained by the May Grunwald-Giemsa technique for microscopic examination. When specialized marrow analyses such as immunophenotyping, flow cytometry, cytogenetic analysis, molecular genetic studies, and microbiology were indicated, additional bone marrow samples were drawn and collected as recommended by the local laboratory.
