# Supplementary figures and images for: Contribution to diagnosis and treatment of bone marrow aspirate results in critically ill patients undergoing bone marrow aspiration: a retrospective study of 193 consecutive patients
Source: J Intensive Care. 2017 Dec 4;5:67. doi: 10.1186/s40560-017-0263-7 (PMC5715543; doi:10.1186/s40560-017-0263-7)

Additional file 3, Annual rates of ICU admissions and adequate bone marrow aspirations


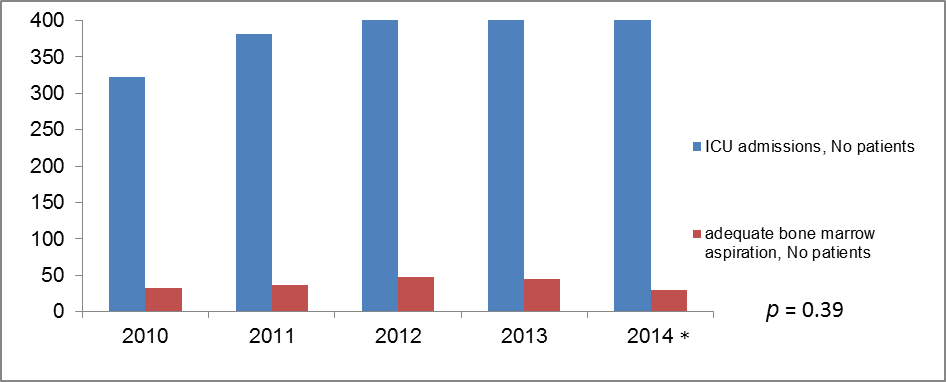


7%

10%

12%

10%

10%

*10 months

Supplement: Supplementary file 3 — Annual rates of ICU admissions and adequate bone marrow aspirations. (DOCX 48 kb) [file 40560_2017_263_MOESM3_ESM.docx]
