# Supplementary material for: Contribution to diagnosis and treatment of bone marrow aspirate results in critically ill patients undergoing bone marrow aspiration: a retrospective study of 193 consecutive patients
Source: J Intensive Care. 2017 Dec 4;5:67. doi: 10.1186/s40560-017-0263-7 (PMC5715543; doi:10.1186/s40560-017-0263-7)
Supplement: Supplementary file 4 — Hematological parameters on the day of bone marrow aspiration in the overall population (N = 193) and in patients with BMA results yielding a CDT (N = 40). (DOCX 12 kb) [file 40560_2017_263_MOESM4_ESM.docx]

Additional file 4, Hematological parameters on the day of bone marrow aspiration in the overall population (N=193) and in patients with BMA results yielding a CDT (N=40).

|  | Overall population (n=193) | Patients with CDT of BMA  (n=40) |
| --- | --- | --- |
| Complete blood count |  |  |
| Hemoglobin level (g/dl)^a^ | 10 [9–11] | 10 [9-10] |
| Leukocyte count (giga/l)^a^ | 10 [7–14] | 6 [2-15] |
| Platelet count (giga/l)^a^ | 90 [53–192] | 70 [51-124] |
| Reticulocytes (giga/l)^a,b^ | 50 [25–84]^b^ | 51 [25-85] |
| Presence of schistocytes^c^ | 13 (31)^b^ | 4 (57)^d^ |
| Presence of abnormal leukocyte differential^c,~~d~~e^ | 48 (25) | 12 (30) |
| Presence of abnormal erythrocytes^c,~~e~~f^ | 5 (3) | 2 (5) |
| Coagulation tests |  |  |
| PT activity (percentage from normal pooled plasma)^a^ | 67 [54–79] | 62 [49-74] |
| aPTT ratio, patient-to-control^a^ | 1.3 [1–1.6] | 1.4 [1.1-1.9] |
| Plasma fibrinogen (g/l)^a^ | 5 [4–7] | 5 [3-6] |

a, median (interquartile range); b, test carried out in 42 patients; c, number of patients and (%); d, test carried out in 7 patients; ~~d~~ e, abnormal leukocyte differential was defined by at least one of the following criteria: lymphopenia <0.750 g/L (n=46), monocytosis >20% of total leukocytes (n=1), eosinophilia >1 g/L or 15% of total leukocytes (n=1); ~~e~~ f, abnormal erythrocytes were defined by at least one of the following criteria: >10 erythroblasts per 100 leukocytes (n=3) or erythrocyte aggregation (n=2); aPTT, activated partial thromboplastin time; PT % activity, prothrombin time as percentage from normal pooled plasma.
