# Supplementary material for: Contribution to diagnosis and treatment of bone marrow aspirate results in critically ill patients undergoing bone marrow aspiration: a retrospective study of 193 consecutive patients
Source: J Intensive Care. 2017 Dec 4;5:67. doi: 10.1186/s40560-017-0263-7 (PMC5715543; doi:10.1186/s40560-017-0263-7)
Supplement: Supplementary file 5 — Hematotoxic agents administered within the 7 days prior to bone marrow aspiration in the 10 patients with maturation arrest of granulocyte precursors observed on marrow aspirates. (DOCX 13 kb) [file 40560_2017_263_MOESM5_ESM.docx]

Additional file 5, Hematotoxic agents administered within the 7 days prior to bone marrow aspiration in the 10 patients with maturation arrest of granulocyte precursors observed on marrow aspirates.

| Patient | Malignancy^a^ | Potentially hematotoxic agent | Contribution to treatment |
| --- | --- | --- | --- |
| 2 | PC | Ceftriaxone | no |
|  |  | Doripenem |  |
| 4 | no | Caspofungin | no |
|  |  | Cefepime |  |
|  |  | Doripenem |  |
|  |  | Levofloxacin |  |
|  |  | Linezolid |  |
| 5 | CLL | Caspofungin | no |
|  |  | Ceftazidime |  |
|  |  | Ciprofloxacin |  |
|  |  | Isoniazid |  |
|  |  | Rifampicin |  |
| 9 | HC | TMP/SMX | Discontinuation of TMP/SMX |
| 17 | no | Ceftriaxone | no |
|  |  | Levofloxacin |  |
|  |  | TMP/SMX |  |
| 25 | no | Tacrolimus | Discontinuation of tacrolimus |
| 37 | no | Cyclophosphamide | Addition of lenogastrim |
| 38 | no | Amoxicillin | Discontinuation of amoxicillin |
| 39 | no | Cefepime | Addition of lenogastrim |
|  |  | Cloxacillin |  |
|  |  | Linezolid |  |
| 40 | AML | Cefepime | no |
|  |  | Cyclosporine |  |
|  |  | Gentamicin |  |
|  |  | Micafungin |  |
|  |  | TMP/SMX |  |
|  |  | Valaciclovir |  |
|  |  | Vancomycin |  |

a, Hematological malignancy or cancer known on admission; AML, acute myeloid leukemia; CLL, chronic lymphocytic leukemia; HC, hepatocellular carcinoma; PC, pulmonary cancer; TMP/SMX, trimethoprim/sulfamethoxazole;
