# Supplementary material for: Contribution to diagnosis and treatment of bone marrow aspirate results in critically ill patients undergoing bone marrow aspiration: a retrospective study of 193 consecutive patients
Source: J Intensive Care. 2017 Dec 4;5:67. doi: 10.1186/s40560-017-0263-7 (PMC5715543; doi:10.1186/s40560-017-0263-7)
Supplement: Supplementary file 6 — Results of the univariate analysis in the overall population (n = 193 patients). (DOCX 14 kb) [file 40560_2017_263_MOESM6_ESM.docx]

Additional file 6, Results of the univariate analysis in the overall population (n=193 patients)

| Variables | No CDT | CDT | *p* |
| --- | --- | --- | --- |
|  | n=153 | n=40 |  |
| Characteristics on ICU admission |  |  |  |
| Age in years^a^ | 67 ± 13 | 63 ± 17 | 0.143 |
| Known hematological malignancy or cancer^b^ | 22 (14) | 11 (28) | 0.05 |
| Known non-malignant hematological abnormality^b^ | 8 (5) | 6 (15) | 0.045 |
| Known hematological malignancy or cancer or non-malignant hematological abnormality^b^ | 30 (20) | 16 (40) | 0.007 |
| Reason for ICU admission^b^ |  |  | 0.597 |
| Acute respiratory failure | 53 (35) | 14 (35) |  |
| Sepsis/septic shock | 36 (24) | 14 (35) |  |
| Acute renal failure | 18 (12) | 4 (10) |  |
| Coma | 15 (10) | 4 (10) |  |
| Cardiac arrest | 9 (6) | 0 (0) |  |
| Thrombotic microangiopathy | 6 (4) | 1 (3) |  |
| Other shock | 7 (5) | 1 (3) |  |
| Metabolic disorder | 4 (3) | 2 (5) |  |
| Post-surgery | 5 (3) | 0 (0) |  |
| Characteristics on the day of BMA |  |  |  |
| Indication for bone marrow examination^b^ |  |  | 0.001 |
| Isolated thrombocytopenia^c^ | 70 (46) | 12 (30) |  |
| Agranulocytosis | 0 (0) | 6 (15) |  |
| Suspected hemophagocytic syndrome | 14 (10) | 4 (10) |  |
| Suspected hematological malignancy | 54 (35) | 17 (43) |  |
| Suspected cancer | 5 (3) | 1 (3) |  |
| Suspected disseminated tuberculosis | 5 (3) | 0 (0) |  |
| Other | 5 (3) | 0 (0) |  |
| Characteristics on the day of BMA |  |  |  |
| Sepsis on the day of bone marrow examination^b^ | 96 (63) | 28 (70) | 0.394 |
| Adenopathy/splenomegaly^b^ | 25 (16) | 7 (18) | 0.861 |
| Monoclonal protein^b^ | 14 (9) | 4 (10) | 0.770 |
| Exposure to potential hematotoxic drug^b,d^ | 136 (89) | 35 (86) | 0.803 |
| Abnormal leukocyte differentials or red blood cells^b^ | 40 (26) | 13 (33) | 0.423 |
| SOFA score^a^ | 8 ± 4 | 10 ± 5 | 0.009 |
| Platelet-count SOFA subscore^a^ | 1 ± 1 | 2 ± 1 | 0.04 |
| Platelet-count SOFA subscore > 0^b^ | 95 (62) | 33 (83) | 0.015 |
| SOFA – platelet-count SOFA subscore^a^ | 6 ± 4 | 8 ± 5 | 0.004 |
| Pre-BMA HScore^e,f^ | 52 [19-82] | 101 [63-146] | <0.001 |

a, mean ± standard deviation; b, number of patients and (percentage); c, thrombocytopenia was the only indication for BMA; d, excluding heparin and antiplatelet agent; e, median and [interquartile range]; f, calculated with no points assigned for the cytological variable; BMA, bone marrow aspiration; CDT, contribution to diagnosis and/or treatment; Hscore, reactive hemophagocytic syndrome diagnostic score; ICU, intensive care unit; SOFA, sequential organ failure assessment.
