# Supplementary material for: Contribution to diagnosis and treatment of bone marrow aspirate results in critically ill patients undergoing bone marrow aspiration: a retrospective study of 193 consecutive patients
Source: J Intensive Care. 2017 Dec 4;5:67. doi: 10.1186/s40560-017-0263-7 (PMC5715543; doi:10.1186/s40560-017-0263-7)
Supplement: Supplementary file 7 — Multivariable analysis of factors associated with a CDT of BMA in the 181 patients without HLH. (DOCX 12 kb) [file 40560_2017_263_MOESM7_ESM.docx]

Additional file 7, Multivariable analysis of factors associated with a CDT of BMA in the 181 patients without HLH

| Variable | Odds ratio | 95% confidence interval | *P* value |
| --- | --- | --- | --- |
| First model | | | |
| Hematological malignancy, cancer or non-malignant hematological abnormality known on admission | 4.46 | [1.68-11.84] | 0.003 |
| Indication of BMA excluding isolated TP^a,b^ | 5.90 | [1.96-17.74] | 0.002 |
| SOFA score^c^ | 1.16 | [1.03-1.29] | 0.012 |
| Pre-BMA HScore^c,d^ | 1.02 | [1-1.03] | 0.012 |
| Second Model |  |  |  |
| Hematological malignancy, cancer or non-malignant hematological abnormality known on admission | 3.84 | [1.36-10.81] | 0.011 |
| Indication of BMA excluding isolated TP^a,b^ | 7.17 | [2.26-22.72] | 0.001 |
| SOFA – platelet-count SOFA subscore^c^ | 1.15 | [1.02-1.30] | 0.026 |
| Pre-BMA HScore^c,d^ | 1.01 | [1-1.03] | 0.023 |
| Platelet count SOFA subscore 0 versus other groups^c^ | 2.38 | [0.78-7.31] | 0.129 |

a, thrombocytopenia may be present or absent in these patients; b, Isolated thrombocytopenia, i.e. thrombocytopenia was the only indication for BMA; c, per point; d, calculated with no points assigned for the cytological variable; BMA, bone marrow aspiration; CDT, contribution to diagnosis and/or treatment; HScore, reactive hemophagocytic syndrome diagnostic score; SOFA, sequential organ failure assessment; TP, thrombocytopenia.
