# Supplementary material for: Contribution to diagnosis and treatment of bone marrow aspirate results in critically ill patients undergoing bone marrow aspiration: a retrospective study of 193 consecutive patients
Source: J Intensive Care. 2017 Dec 4;5:67. doi: 10.1186/s40560-017-0263-7 (PMC5715543; doi:10.1186/s40560-017-0263-7)
Supplement: Supplementary file 8 — Results of the univariate analysis in the 160 patients without hematological malignancy or cancer known on admission. (DOCX 14 kb) [file 40560_2017_263_MOESM8_ESM.docx]

Additional file 8, Results of the univariate analysis in the 160 patients without hematological malignancy or cancer known on admission

| Variables | No CDT | CDT | *p* |
| --- | --- | --- | --- |
|  | n=131 | n=29 |  |
| Characteristics on ICU admission |  |  |  |
| Age in years^a^ | 67 ± 13 | 62 ± 18 | 0.144 |
| Known non-malignant hematological abnormality^b^ | 8 (6) | 5 (17) | 0.047 |
| Reason for ICU admission^b^ |  |  | 0.260 |
| Acute respiratory failure | 46 (35) | 8 (26) |  |
| Sepsis/septic shock | 29 (22) | 12 (41) |  |
| Acute renal failure | 16 (12) | 2 (7) |  |
| Coma | 14 (11) | 4 (14) |  |
| Cardiac arrest | 8 (6) | 0 (0) |  |
| Thrombotic microangiopathy | 4 (3) | 0 (0) |  |
| Other shock | 6 (5) | 1 (3) |  |
| Metabolic disorder | 3 (2) | 2 (7) |  |
| Post-surgery | 5 (4) | 0 (0) |  |
| Characteristics on the day of BMA |  |  |  |
| Indication for bone marrow examination^b^ |  |  | 0.001 |
| Isolated thrombocytopenia^c^ | 59 (45) | 11 (38) |  |
| Agranulocytosis | 0 (0) | 6 (21) |  |
| Suspected hemophagocytic syndrome | 12 (9) | 3 (10) |  |
| Suspected hematological malignancy | 48 (37) | 8 (28) |  |
| Suspected cancer | 3 (3) | 1 (3) |  |
| Suspected disseminated tuberculosis | 4 (3) | 0 (0) |  |
| Other | 5 (4) | 0 (0) |  |
| Characteristics on the day of BMA |  |  |  |
| Sepsis on the day of bone marrow examination^b^ | 84 (64) | 19 (66) | 0.887 |
| Adenopathy/splenomegaly^b^ | 23 (18) | 6 (21) | 0.692 |
| Monoclonal protein^b^ | 13 (10) | 3 (10) | 1.000 |
| Exposure to potential hematotoxic drug^b,d^ | 116 (89) | 25 (86) | 0.799 |
| Abnormal leukocyte differentials or red blood cells^b^ | 31 (24) | 7 (24) | 0.957 |
| SOFA score^a^ | 8 ± 4 | 11 ± 5 | 0.01 |
| Platelet-count SOFA subscore^a^ | 1 ± 1 | 2 ± 1 | 0.12 |
| Platelet-count SOFA subscore > 0^b^ | 78 (60) | 23 (79) | 0,046 |
| SOFA – platelet-count SOFA subscore^a^ | 6 ± 4 | 9 ± 5 | 0.003 |
| Pre-BMA HScore^e,f^ | 52 [19–81] | 98 [63–140] | <0.001 |

a, mean ± standard deviation; b, number of patients and (percentage); c, thrombocytopenia was the only indication for BMA; d, excluding heparin and antiplatelet agent; e, median and [interquartile range]; f, calculated with no points assigned for the cytological variable; BMA, bone marrow aspiration; CDT, contribution to diagnosis and/or treatment; Hscore, reactive hemophagocytic syndrome diagnostic score; ICU, intensive care unit; SOFA, sequential organ failure assessment.
